# Editorial: The role of neurogenesis in cognitive decline during aging

**DOI:** 10.3389/fgene.2026.1861909

**Published:** 2026-05-07

**Authors:** Yu-zhang Liu, Kristy A. Nielson, Cathy W. Levenson

**Affiliations:** 1 Department of Neuroscience, University of Pittsburgh, Pittsburgh, PA, United States; 2 Department of Psychiatry, University of Pittsburgh, Pittsburgh, PA, United States; 3 Department of Psychology, Marquette University, Milwaukee, WI, United States; 4 Department of Biomedical Sciences, Florida State University College of Medicine, Tallahassee, FL, United States

**Keywords:** aging, biomarkers, cognitive decline, neurobiological mechanisms, therapeutic strategies

Aging has been one of the most significant population trends in the 21st century (United Nations Population Fund, 2012). According to the report by the World Health Organization (WHO), one out of six people in the world will be 60 years old or over by 2030 (World Health Organization, 2025). With longer life expectancy, decreased birth rate, and increasing migration, the trend toward population aging that was first identified in high-income countries is now common also in developing countries. Thus, there is an urgent global need to adapt to population aging and contribute to building an age-friendly society.

Much of the responsibility for adjusting to the aging of society will fall on scientific researchers and medical workers. As the population ages and cognitive decline become increasingly prevalent, there will be increasing urgency for a deeper understanding of the underlying mechanisms of cognitive change and for pioneering and implementing effective strategies to mitigate cognitive impairments. Cognitive decline progresses along a spectrum, beginning with changes observed during normal aging, advancing to clinically defined cognitive impairment such as mild cognitive impairment (MCI), and becoming more pronounced following acute pathological insults such as strokes and neurodegenerative disease. The causes of the cognitive deterioration have been investigated at multiple levels (Mosti et al., 2019), from cortical gray matter shrinkage (Taki et al., 2011), reduced cerebral white matter integrity (Marstaller et al., 2015), vascular dysfunction (Toth et al., 2017), to neuroinflammation (Sun et al., 2014), the emergence of neurofibrillary plaques and tangles (Green et al., 2000), reduced dendritic length and branching (Dickstein et al., 2007), and diminished neurogenesis in the hippocampus (Lazarov et al., 2010).

While understanding the underlying mechanisms of cognitive decline is essential, it is equally important to translate these insights into strategies that can prevent and mitigate cognitive impairments. Increasing efforts have therefore been directed toward identifying effective interventions to preserve cognitive function across aging and disease. Among these, exercise has emerged as one of the most extensively studied and promising non-pharmacological strategies, as exercise has consistently demonstrated beneficial effects on cognitive function, supported by its ability to enhance cerebral blood flow (Alfini et al., 2019), promote neuroplasticity (Lista and Sorrentino, 2010), and reduce neuroinflammation (Hu et al., 2024). On the other hand, pharmacological approaches are gaining increasing attention. Acetylcholinesterase-inhibitors (Kim et al., 2020), Ginkgo biloba (Zhang et al., 2016), non-steroidal anti-inflammatory drugs (Wang et al., 2016), hormone replacement therapy (Labrie et al., 1998), and nootropic compounds (Froestl et al., 2012) are among the most extensively studied treatments for cognitive impairment, including potential nootropic compounds such as cannabidiol (CBD) treatments (Ortiz et al., 2023). Although these treatments have yielded mixed outcomes, they provided valuable insights into the molecular and cellular pathways underlying cognitive function.

We selected three Original Research Articles and two Literature Reviews for this Research Topic. From fundamental research in animal models (rodents) to clinical therapeutic interventions with patients, these five articles show readers the latest progress scientists have made, and how much of the recent studies have been translated to clinical approaches, to help people prevent, predict, and treat cognitive decline.

In the Research Article from Ai et al., the authors offered clinical evidence bridging glymphatic function, perivascular space (PVS), and cognitive performance in patients with cerebral small vessel disease (CSVD) using diffusion tensor imaging analysis along the perivascular space (DTI-ALPS). They reported that the CSVD patients showed decreased analysis along the perivascular space index in some brain regions, which negatively correlated with perivascular space volume fraction, and positively correlated with physical and cognitive functions, providing a potential non-invasive avenue for early detection of cognitive decline.

In the Research Article from Mirza Agha et al., CBD treatment effects were measured relative to cognitive decline in aged mice with a combination of behavioral tests and immunohistochemical approaches. The results revealed that CBD-treated aged mice showed improved performance on object and spatial memory mediated by the perirhinal cortex and hippocampus. CBD was also shown to reduce inflammation in the hippocampus and medial prefrontal cortex. Moreover, they did not detect negative side effects after long-term application of CBD, such as changes in hippocampal volume or impairments in learning and memory functions. They concluded that CBD may be a potential clinical treatment for age-related cognitive impairment.

In the third Research Article by Wang et al., the authors investigated brain functional connectivity alterations in patients with asymptomatic carotid stenosis (ACS) using resting-state functional-MRI and dynamic connectivity analyses. Compared to healthy controls, ACS patients exhibited widespread changes in both static and dynamic functional connectivity across multiple large-scale networks, including fronto-parietal and salience networks. Notably, temporal variability in network activity correlated with cognitive performance and memory. These findings are significant as they suggest that ACS-related cognitive decline is associated with both disrupted network organization and altered temporal dynamics of brain connectivity.

In a Review Article, Zhu et al. summarized current evidence supporting aerobic exercise as a promising non-pharmacological intervention for post-stroke cognitive impairment. The review integrates clinical and preclinical studies, showing that aerobic exercise improves multiple cognitive domains and functional outcomes. This review is a significant addition to the literature because it mechanistically links these benefits to enhanced cerebral blood flow and increased neuroplasticity via brain-derived neurotrophic factor (BDNF) signaling and reduced neuroinflammation and oxidative stress. The authors also highlighted variability in optimal exercise protocols and emphasized the need for individualized, mechanism-driven rehabilitation strategies in the clinical environment.

Finally, the Review Article by Ma et al. provided an overview of cognitive reserve, brain maintenance, and brain reserve, which was recently renamed “resilience” (Stern et al., 2023), to explain the brain’s ability to withstand damage caused by aging or pathology. They highlighted the molecular and cellular mechanisms of glial cells and the fundamental roles they play in Alzheimer’s disease (AD), as well as in therapeutic development. Last but not least, this work makes a significant contribution by providing a list of biomarkers that have been recently identified and may eventually contribute to early detection of AD in the resilience framework, to potentially be applied as individualized treatments.

The Research Topic assembled here aimed to address the questions posed regarding the underlying causes of age-related cognitive decline and, importantly, strategies for early diagnosis and delayed disease progression. Together, these studies not only consolidate current knowledge across molecular, systematic, and clinical levels, but also point toward emerging research directions and translational opportunities. They advance our understanding of key mechanisms, including glymphatic function, network-level brain dynamics, and resilience frameworks underlying cognitive decline. In parallel, they highlight both pharmacological (e.g., CBD) and non-pharmacological (e.g., aerobic exercise) interventions as promising therapeutic strategies. Importantly, these findings open the door to future longitudinal studies, biomarker-guided diagnostics, and targeted therapeutic trials, ultimately advancing the goal of mitigating cognitive decline in aging populations.
